# Correction: Structure-guided microbial targeting of antistaphylococcal prodrugs

**DOI:** 10.7554/eLife.75743

**Published:** 2021-11-25

**Authors:** Justin J Miller, Ishaan T Shah, Jayda Hatten, Yasaman Barekatain, Elizabeth A Mueller, Ahmed M Moustafa, Rachel L Edwards, Cynthia S Dowd, Geoffrey C Hoops, R Jeremy Johnson, Paul Planet, Florian L Muller, Joseph Jez, Audrey R Odom John

**Keywords:** Other

2021. Miller JJ, Shah IT, Hatten J, Y Barekatain, Mueller EA, Moustafa AM, Edwards RL,Dowd CS, Hoops CJ, Johnson RJ, Planet PJ, Muller FL, Jez JM, Odom John AR. Structure-guided microbial targeting of antistaphylococcal prodrugs. eLife **10**:e66657. doi: 10.7554/eLife.66657

Published 19 July 2021

In our manuscript we utilized a fluorescent ester substrate library to characterize the substrate specificity of esterases from human and mouse serum, the staphylococcal esterases FrmB and GloB, and the activity of whole-cell *Staphylococcus aureus*. While the generation of this library has been described and was cited (White et al. 2018 JBC), the materials of this library are inherently limited, and the generation of this library required substantially intellectual contributions on the part of Prof. Geoffrey C Hoops and Prof. R Jeremy Johnson. We are therefore formally correcting the eLife paper to include both as co-authors on this paper. The figure reporting the structures of this library (Figure 3—figure supplement 1) was derived from an original figure provided by Dr. Hoops. In addition, both Dr. Hoops and Dr. Johnson have reviewed the manuscript for accuracy, have access to primary (raw) data as needed, support the conclusions of this manuscript, and are responsible for all its parts.

The details of Profs. Hoops and Johnson’s contribution are explained below:

“Professors Hoops and Johnson first derived and employed the fluorescent ester substrate library as a means of efficiently characterizing esterase substrate specificity and promiscuity. Creation of this library was central to our manuscript and enabled the characterization of human and mouse serum esterases, staphylococcal esterases FrmB and GloB, and whole cell *Staphylococcus aureus*.”

**New authors list:**

Justin J Miller, Ishaan T Shah, Jayda Hatten, Yasaman Barekatain, Elizabeth A Mueller, Ahmed M Moustafa, Rachel L Edwards, Cynthia S Dowd, **Geoffrey C Hoops, R Jeremy Johnson,** Paul J Planet, Florian L Muller, Joseph M Jez, Audrey R Odom John

**Original authors list:**

Justin J Miller, Ishaan T Shah, Jayda Hatten, Yasaman Barekatain, Elizabeth A Mueller, Ahmed M Moustafa, Rachel L Edwards, Cynthia S Dowd, Paul J Planet, Florian L Muller, Joseph M Jez, Audrey R Odom John

**Details for the omitted authors:**

**Geoffrey C Hoops**

Department of Chemistry and Biochemistry, Butler University, Indianapolis, Indiana 46208–3443

Contribution: Methodology

Competing interests: No competing interests exist

**R Jeremy Johnson**

Department of Chemistry and Biochemistry, Butler University, Indianapolis, Indiana 46208–3443

Contribution: Methodology

Competing Interests: No competing interests exist

**Original Acknowledgements:**

We are grateful to the students of the Spring 2020 Biol 4,522 course at Washington University for the creation of FrmB point mutation plasmids and the purification of FrmB point mutants. Thank you to Vandna Kukshal and Jason Schaffer for helpful discussions around data and mechanisms, to the Bubeck-Wardenburg laboratory for sharing benchspace during revisions, and to Petra Levin for assistance with microfluidics and microscopy. Fluorescent ester compounds were generously provided by the laboratory of Geoffrey Hoops (Butler University). AOJ is supported by NIH/NIAID R01-AI103280, R21-AI123808, and R21-AI130584, and AOJ is an Investigator in the Pathogenesis of Infectious Diseases (PATH) of the Burroughs Wellcome Fund. This publication was made possible in part by Grant Number UL1 RR024992 from the NIH-National Center for Research Resources (NCRR).

**Revised Acknowledgements:**

We are grateful to the students of the Spring 2020 Biol 4,522 course at Washington University for the creation of FrmB point mutation plasmids and the purification of FrmB point mutants. Thank you to Vandna Kukshal and Jason Schaffer for helpful discussions around data and mechanisms, to the Bubeck-Wardenburg laboratory for sharing benchspace during revisions, and to Petra Levin for assistance with microfluidics and microscopy. AOJ is supported by NIH/NIAID R01-AI103280, R21-AI123808, and R21-AI130584, and AOJ is an Investigator in the Pathogenesis of Infectious Diseases (PATH) of the Burroughs Wellcome Fund. This publication was made possible in part by Grant Number UL1 RR024992 from the NIH-National Center for Research Resources (NCRR). RJJ was supported by a grant from the National Institutes of Health (R15-GM110641).

The article has been corrected accordingly.

