# Supplementary material for: Structure-guided microbial targeting of antistaphylococcal prodrugs
Source: eLife. 2021 Jul 19;10:e66657. doi: 10.7554/eLife.66657 (PMC8318587; doi:10.7554/eLife.66657)
Supplement: Supplementary file 1. [file elife-66657-supp1.docx]

**Supplementary File 1** Primers used during this study.

| No | Name | Sequence | Use |
| --- | --- | --- | --- |
| 1 | NWMN_0144_F | TTTTCCTGATCCTGATTCAC | Sanger Sequencing |
| 2 | NWMN_0144_R | ATGATGCTTCCATGTTTGTT | Sanger Sequencing |
| 3 | NWMN_0306_F | AATACACCGGGTAACACAAC | Sanger Sequencing |
| 4 | NWMN_0306_R | CGTTTTGTTGAGCTAATTCC | Sanger Sequencing |
| 5 | NWMN_0309_F | ACCATGCTTAAAGGGATTTT | Sanger Sequencing |
| 6 | NWMN_0309_R | TGTCACCTAAGTCAACACCA | Sanger Sequencing |
| 7 | NWMN_0407 (*lpl4nm*) _F | CCGTTGGAGATAGGAAGTTA | Sanger Sequencing |
| 8 | NWMN_0407 (*lpl4nm*) _R | TTTGTGCTTCTTTTGAACCT | Sanger Sequencing |
| 9 | NWMN_0654_F | GAAAATGGAAGACTGATTGC | Sanger Sequencing |
| 10 | NWMN_0654_R | TAATGCATCTGACAAAGTCG | Sanger Sequencing |
| 11 | NWMN_0762_F | GGTGAAGTTTTGGACGATAA | Sanger Sequencing |
| 12 | NWMN_0762_R | TTTTCATCTGTCCGACTTTT | Sanger Sequencing |
| 13 | NWMN_1101_F | TCCACCTATTGGAATTATCG | Sanger Sequencing |
| 14 | NWMN_1101_R | AGACGTTCAATTTCAGTGCT | Sanger Sequencing |
| 15 | NWMN_1192 (*pgsA*) _F | TGGGACGAAGTAATTACAGTT | Sanger Sequencing |
| 16 | NWMN_1192 (*pgsA*) _R | ATATCCCCCTTGTATCGTTT | Sanger Sequencing |
| 17 | NWMN_1308 (*dapD*) _F | TCTATTCGTGGAGGTACGAT | Sanger Sequencing |
| 18 | NWMN_1308 (*dapD*) _R | ATCGTATGTGAGCCATTACC | Sanger Sequencing |
| 19 | NWMN_1410_F | CGATAAACCTAAACCACTCG | Sanger Sequencing |
| 20 | NWMN_1410_R | ATAAACAATGCTTGCCAAAT | Sanger Sequencing |
| 21 | NWMN_1505_F | TGAAGGTGAATTAAGCGATG | Sanger Sequencing |
| 22 | NWMN_1505_R | TGCTATTCCCAATTTGTTCA | Sanger Sequencing |
| 23 | NWMN_1655_F | GAATTGTTGCAATTTAATGGT | Sanger Sequencing |
| 24 | NWMN_1655_R | AACGTAATCATGCTCCATTC | Sanger Sequencing |
| 25 | NWMN_1679_F | CCATGGGAAAAATTAGACAA | Sanger Sequencing |
| 26 | NWMN_1679_R | AAATATCGCCTCACCTTTTT | Sanger Sequencing |
| 27 | NWMN_1723 (*hemY*) _F | GCCGAATACACATCCATTAT | Sanger Sequencing |
| 28 | NWMN_1723 (*hemY*) _R | AACCTTTGTCTCTGCTTCAA | Sanger Sequencing |
| 29 | NWMN_1851 (*nadC*) _F | AGCCATTTTAGCACCATAAA | Sanger Sequencing |
| 30 | NWMN_1851 (*nadC*)_R | TAGAATCCTGTCCTCCTGAA | Sanger Sequencing |
| 31 | NWMN_2057 (*mtlF*)_F | TGTACAACGGTGTTGTTTTG | Sanger Sequencing |
| 32 | NWMN_2057 (*mtlF*)_R | CGGTGAATAGTACGAGAGGA | Sanger Sequencing |
| 33 | NWMN_2528_F | ACTGATGCTTTACCAGAAAC | Sanger Sequencing |
| 34 | NWMN_2528_R | TCAGCGGTAGTAATAAAGGT | Sanger Sequencing |
