# Supplementary material for: Structure-guided microbial targeting of antistaphylococcal prodrugs
Source: eLife. 2021 Jul 19;10:e66657. doi: 10.7554/eLife.66657 (PMC8318587; doi:10.7554/eLife.66657)
Supplement: Transparent reporting form [file elife-66657-transrepform.docx]

***eLife’s* transparent reporting form**

We encourage authors to provide detailed information *within their submission* to facilitate the interpretation and replication of experiments. Authors can upload supporting documentation to indicate the use of appropriate reporting guidelines for health-related research (see [EQUATOR Network](http://www.equator-network.org/%20)), life science research (see the [BioSharing Information Resource](https://biosharing.org/)), or the [ARRIVE guidelines](http://www.plosbiology.org/article/info:doi/10.1371/journal.pbio.1000412) for reporting work involving animal research. Where applicable, authors should refer to any relevant reporting standards documents in this form.

If you have any questions, please consult our Journal Policies and/or contact us: [editorial@elifesciences.org](mailto:editorial@elifesciences.org).

**Sample-size estimation**

- You should state whether an appropriate sample size was computed when the study was being designed
- You should state the statistical method of sample size computation and any required assumptions
- If no explicit power analysis was used, you should describe how you decided what sample (replicate) size (number) to use

A sample size of 3 was applied for all experiments. In some cases due to difficulty in acquiring sample (fresh human sera) sample size was decreased to 2 with technical replicates in place. In microscopy experiments all cells visible in the frame were included. Sample sizes may be found in the figure legends as well as in the methods section.

Please outline where this information can be found within the submission (e.g., sections or figure legends), or explain why this information doesn’t apply to your submission:

**Replicates**

- You should report how often each experiment was performed
- You should include a definition of biological versus technical replication
- The data obtained should be provided and sufficient information should be provided to indicate the number of independent biological and/or technical replicates
- If you encountered any outliers, you should describe how these were handled
- Criteria for exclusion/inclusion of data should be clearly stated
- High-throughput sequence data should be uploaded before submission, with a private link for reviewers provided (these are available from both GEO and ArrayExpress)

Please outline where this information can be found within the submission (e.g., sections or figure legends), or explain why this information doesn’t apply to your submission:

Information about experimental replicates is provided within figure legends and the methods section. Biological replicates are defined as: independent colonies (for sequencing) or separate days (for biological and enzymological experiments) with fresh materials acquired on the start of each day. Technical replicates are experiments performed at the same time as one another, though in separate wells to account for variation in pipetting or instrumentation. Outliers were found when we auto-identified regions of interest in our microscopy experiments, however when those regions were visually inspected they were found to be debris instead of cells and excluded. Sequencing data and structure data is provided in the manuscript and deposited on the NCBI BioProject database (PRJNA648156) or in the protein data bank (FrmB- 7L0A, GloB- 7L0B).

**Statistical reporting**

- Statistical analysis methods should be described and justified
- Raw data should be presented in figures whenever informative to do so (typically when N per group is less than 10)
- For each experiment, you should identify the statistical tests used, exact values of N, definitions of center, methods of multiple test correction, and dispersion and precision measures (e.g., mean, median, SD, SEM, confidence intervals; and, for the major substantive results, a measure of effect size (e.g., Pearson's r, Cohen's d)
- Report exact p-values wherever possible alongside the summary statistics and 95% confidence intervals. These should be reported for all key questions and not only when the p-value is less than 0.05.

Please outline where this information can be found within the submission (e.g., sections or figure legends), or explain why this information doesn’t apply to your submission:

Raw data is displayed in all cases except for when presentation of the raw data is overwhelmingly unclear. Statistical analyses and exact replicate and sample size information are described in figure legends.

(For large datasets, or papers with a very large number of statistical tests, you may upload a single table file with tests, Ns, etc., with reference to sections in the manuscript.)

**Group allocation**

- Indicate how samples were allocated into experimental groups (in the case of clinical studies, please specify allocation to treatment method); if randomization was used, please also state if restricted randomization was applied
- Indicate if masking was used during group allocation, data collection and/or data analysis

Please outline where this information can be found within the submission (e.g., sections or figure legends), or explain why this information doesn’t apply to your submission:

Masking was not applied in this case as all methods were quantitative. Sample allocation was not necessary as sample quantity was sufficient so as to split it and have all samples undergo treatment and control. Randomization was used to decide the order of sample run on a given day.

**Additional data files (“source data”)**

- We encourage you to upload relevant additional data files, such as numerical data that are represented as a graph in a figure, or as a summary table
- Where provided, these should be in the most useful format, and they can be uploaded as “Source data” files linked to a main figure or table
- Include model definition files including the full list of parameters used
- Include code used for data analysis (e.g., R, MatLab)
- Avoid stating that data files are “available upon request”

Please indicate the figures or tables for which source data files have been provided:

All figures and tables have their source data files provided as exact values in supplemental tables.
